# Characterization of the surfaceome of the metal-reducing bacterium *Desulfotomaculum reducens*

**DOI:** 10.3389/fmicb.2014.00432

**Published:** 2014-08-19

**Authors:** Elena Dalla Vecchia, Paul P. Shao, Elena Suvorova, Diego Chiappe, Romain Hamelin, Rizlan Bernier-Latmani

**Affiliations:** ^1^Environmental Microbiology Laboratory, Environmental Engineering Institute, École Polytechnique Fédérale de LausanneLausanne, Switzerland; ^2^Proteomics Core Facility, Core Facility PTECH, École Polytechnique Fédérale de LausanneLausanne, Switzerland

**Keywords:** Fe(III) reduction, *Desulfotomaculum reducens*, surfaceome, extracellular electron transfer, Gram-positive bacteria, cell-wall protein, membrane protein

## Abstract

*Desulfotomaculum reducens* strain MI-1 is a Gram-positive, sulfate-reducing bacterium also capable of reducing Fe(III). Metal reduction in Gram-positive bacteria is poorly understood. Here, we investigated Fe(III) reduction with lactate, a non-fermentable substrate, as the electron donor. Lactate consumption is concomitant to Fe(III) reduction, but does not support significant growth, suggesting that little energy can be conserved from this process and that it may occur fortuitously. *D. reducens* can reduce both soluble [Fe(III)-citrate] and insoluble (hydrous ferric oxide, HFO) Fe(III). Because physically inaccessible HFO was not reduced, we concluded that reduction requires direct contact under these experimental conditions. This implies the presence of a surface exposed reductase capable of transferring electrons from the cell to the extracellular electron acceptor. With the goal of characterizing the role of surface proteins in *D. reducens* and of identifying candidate Fe(III) reductases, we carried out an investigation of the surface proteome (surfaceome) of *D. reducens*. Cell surface exposed proteins were extracted by trypsin cell shaving or by lysozyme treatment, and analyzed by liquid chromatography-tandem mass spectrometry. This investigation revealed that the surfaceome fulfills many functions, including solute transport, protein export, maturation and hydrolysis, peptidoglycan synthesis and modification, and chemotaxis. Furthermore, a few redox-active proteins were identified. Among these, three are putatively involved in Fe(III) reduction, i.e., a membrane-bound hydrogenase 4Fe-4S cluster subunit (Dred_0462), a heterodisulfide reductase subunit A (Dred_0143) and a protein annotated as alkyl hydroperoxide reductase but likely functioning as a thiol-disulfide oxidoreductase (Dred_1533).

## Introduction

The cell structure of Gram-positive bacteria is characterized by a single membrane surrounded by a relatively thick cell wall (CW). The CW's main component is peptidoglycan (PG), consisting of polysaccharide layers of alternating N-acetylmuramic acid (NAM) and N-acetylglucosamine (NAG) monomers, cross-linked through oligopeptide tails. In addition to PG, other polymers, i.e., teichoic, lipoteichoic, teichuronic acids, are found in the Gram-positive CW (Navarre and Schneewind, 1999; Desvaux et al., 2006; Weidenmaier and Peschel, 2008). The CW compact structure has a fundamental structural role, since it provides rigidity to the cells and protects them from osmotic lysis. However, this cell layer is also responsible for mediating all interactions with the surrounding environment by means of the proteins displayed on its surface (Navarre and Schneewind, 1999). The ensemble of surface-exposed proteins constitutes the so-called *surfaceome* of a bacterium and fulfills a variety of functions (Cullen et al., 2005; Desvaux et al., 2006). One of the most relevant, and most studied, roles of surface proteins is in virulence and host interaction in pathogenic strains (Navarre and Schneewind, 1999; Cabanes et al., 2002; Nandakumar et al., 2005; Marraffini et al., 2006). Other important roles for surface proteins include: adhesion to substrates and intercellular interaction for biofilm formation; environmental signal reception and activation of a response (e.g., chemotaxis, stress response); motility (flagella); intercellular genetic exchange (i.e., conjugation through pili); extracellular substrate binding for transport to the cytoplasm; CW digestion, either self-directed as in the case of autolysins, or aimed at cells of other species as a defense mechanism (Smith et al., 2000; Desvaux et al., 2006). Another role most likely played by surface proteins of Gram-positive bacteria is extracellular electron transfer. In particular, Carlson et al. identified a surface-exposed multi-heme *c*-type cytochrome that is putatively involved in extracellular Fe(III) reduction in *Thermincola potens* JR (Carlson et al., 2012).

Proteins of the surfaceome accomplish their functions via the exposure of their catalytic domain to the extracellular environment. There are several binding mechanisms that allow proteins to be partially surface exposed. Membrane-associated proteins are anchored to the membrane through one or multiple transmembrane helices (TMHs), and lipoproteins are covalently anchored to the long-chain fatty acids of the cytoplasmic membrane (CM). Also, CW-associated proteins are covalently attached to the cell wall through a conserved LPxTG domain. Proteins not covalently associated to the cell wall are attached through binding domains such as CWBD_1, CWBD_2, LysM-type and GW-type (Navarre and Schneewind, 1994; Desvaux et al., 2006). CWBD_1 binds the protein to the choline residues of teichoic and lipoteichoic acids, while the CW component recognized by CWBD_2 is, as of yet, unidentified; LysM-type domain binds directly to the PG; GW-type domains contain glycine-tryptophane modules and are responsible for protein binding to lipoteichoic acids (Cabanes et al., 2002; Desvaux et al., 2006). Another CW-association domain is the S-layer homology domain (SLHD), which is found in proteins that form another surface layer (S-layer) completely surrounding the cell wall of certain Gram-positive bacteria.

In this work, we undertook a proteomic investigation of the surfaceome of *Desulfotomaculum reducens* MI-1. Our interest in this bacterium and its surface-exposed proteins is motivated by the dearth of attention given to the surfaceome of environmental Gram-positive bacterial strains because of the priority given to pathogenic species. Additionally, *D. reducens* is able to reduce Fe(III), in soluble and insoluble form, with a non-fermentable substrate, i.e., lactate, as an electron donor. As part of this study, we found that direct surface contact is necessary for *D. reducens* cells to be able to transfer electrons to the extracellular electron acceptor. Thus, we investigated the surfaceome of *D. reducens* in an attempt to identify the electron transport chain that allows reducing power to be conveyed from the cytoplasm, across the CM and the CW, to the terminal electron acceptor (TEA).

## Materials and methods

### Organism and growth conditions

*D. reducens* strain MI-1 was grown anaerobically in basal Widdel Low Phosphate (WLP) medium amended with trace elements and vitamins (Bernier-Latmani et al., 2010) at pH 7.1 ± 0.1. WLP medium amended with 0.05% yeast extract (Becton, Dickinson & Company, Sparks, MD, USA), 30 mM NaHCO_3_ (Acros, Geel, Belgium) and 20 mM pyruvate (pyruvic acid 98%, Acros, Geel, Belgium) was used for fermentative growth. Cells grown at 37°C under these conditions were harvested at late exponential phase by centrifugation at 8000 × g for 15 min (Avanti centrifuge with JLA 91000 or JA-12 rotors, Beckman Coulter, USA), washed in WLP basal medium and used as inoculum (10%) for Fe(III)-reduction experiments. For cell shaving and protoplast formation experiments, all the harvested biomass was transferred to fresh medium. Under all conditions, cells were cultured in serum bottles sealed with blue butyl rubber stoppers and aluminum crimp seals.

For soluble or solid-phase Fe(III) reduction experiments, WLP medium was amended with 10 mM Fe(III)-citrate (Sigma, St. Louis, MO, USA) or hydrous ferric oxide (prepared according to Lovley and Phillips, 1986), 10 mM NaHCO_3_, 0.05% yeast extract and 10 mM of lactate (lactic acid 90%, Acros, Geel, Belgium) as an electron donor. Cultures were incubated at 37°C and sampled for the concentrations of Fe(II), electron donor and the products of electron donor oxidation, as well as for cell growth. Sampling was performed in an anaerobic chamber (Coy, Grass Lake, MI, USA) with an atmosphere of 2.5–3.5% hydrogen (balance nitrogen) with disposable syringes. For the RNA and the surfaceome extraction experiments, only Fe(III)-citrate reduction with lactate and pyruvate fermenting cultures were used, and the former condition was sampled only for Fe(II) concentration. Experimental obstacles hindered us from performing these experiments with HFO, and Fe(III)-citrate was used as an extracellular electron acceptor instead.

For lysed or killed cell preparations, the following protocol was used. A late exponential-phase fermentation culture was harvested by centrifugation (8000 × g for 15 min), washed in WLP basal medium and concentrated 10-fold. Half the cells were lysed by sonication on ice (10 cycles of 5 sonication pulses of 5 s), and subsequently filter-sterilized (0.2 μm filter) to remove residual whole cells. The remaining half cell-concentrate was treated with formaldehyde, to a final concentration of 18%, and incubated overnight at room temperature. After incubation the cells were washed twice to remove the formaldehyde. Killed cells and cell lysate were added to WLP medium amended with HFO and lactate, as for the vegetative cells Fe(III)-reduction experiments.

### HFO-embedded in glass reduction assays

Hydrous ferric oxide (HFO)-embedded glass particles, henceforth referred to as glass-HFO, were prepared and characterized as previously described (Dalla Vecchia et al., 2014).

Reduction assays of glass-HFO by *D. reducens* were carried out similarly to the other Fe(III) reduction experiments: WLP medium was amended with ~4 mM Fe(III), in the form of glass-HFO, 0.05% yeast extract, 10 mM NaHCO_3_, 10 mM lactate, and was inoculated with 10% fermentatively grown, washed, *D. reducens* cells. Positive control reduction experiments with free HFO suspensions (not glass-embedded) were carried out. Samples for total extractable Fe(II) were collected over time.

### AQDS reduction in spent medium

Spent medium was obtained by filter-sterilization (0.2 μm pore size Filtropur S/S filters, Sarstedt, Nümbrecht, Germany) from HFO-reducing cultures. The spent medium was amended with 1 mM anthraquinone disulfonate (AQDS, anthraquinone-2,6-disulfonic acid, disodium salt 90%, Acros). Abiotic reduction of AQDS in the spent media was tested spectrophotometrically by measuring absorbance at 326 nm (A_326_): AQDS is often used as a probe for extracellular electron transfer, since its reduced product is easily identifiable (Bucking et al., 2012).

### Analytical techniques

Growth was quantified by protein content in the case of soluble Fe(III) reduction. A 0.5 ml aliquot of culture was measured with the Qbit protein assay kit and a Qbit Fluorometer (Invitrogen, Zug, Switzerland) according to the supplier's protocol. In the case of HFO reduction, biomass was quantified by direct counting of DAPI-stained cells (Vectashield, Burlingame, CA, USA) by epifluorescence microscopy (Eclipse E800, Nikon, Egg, Switzerland).

Lactate consumption and acetate formation were measured by ion chromatography (DX-3000, Dionex, Sunnyvale, CA) with an IonPac AS11-HC column. Elution was carried out using a gradient of 0.5–30 mM KOH.

A small volume (0.1 mL) of filtered (0.2 μm pore size, PTFE filters, BGB, Geneva, Switzerland) or unfiltered samples were diluted in 0.9 ml of 0.5 M HCl, for soluble Fe(II), or 2 M HCl, for total extractable Fe(II), respectively. Fe(II) was measured according to the ferrozine assay as previously described (Dalla Vecchia et al., 2014).

### qRT-PCR

Reverse transcription- and comparative quantitative-polymerase chain reaction (RT-PCR and qPCR, respectively) were performed on total RNA extracted from *D. reducens* cultures during Fe(III)-citrate reduction with lactate and during pyruvate fermentation. The cultures (25 ml during fermentation, 50 ml during Fe(III) reduction because of the lower cell yield) were harvested after 0.7–1 mM Fe(III) was reduced, or in mid-exponential phase in the case of fermentative growth. Cells were resuspended in 400 μl of 3 mg/ml lysozyme in TE buffer (pH 8.0) and mixed by vortexing. Cells were digested for 10 min at room temperature and amended with 1.4 ml of Buffer RTL (Qiagen) containing freshly added 1% vol/vol ß-mercaptoethanol (Applichem, Damstadt, Germany). After vigorous vortexing, the homogenized cell lysates were stored at −80°C. For further processing, the samples were thawed for 15 min at 37°C in a water bath to dissolve salts.

RNA was extracted using the RNeasy extraction kit (Qiagen, Hilden, Germany) with a double DNase treatment (Qiagen and RQ1, Promega, Madison, WI, USA), as described in detail in Dalla Vecchia et al. (2014). The quantity and quality of the RNA extracted were evaluated with a Nanodrop spectrophotometer (Nanodrop Technologies, Wilmington, DE, USA). First-strand synthesis was carried out using ImProm-II Reverse Transcriptase (Promega) and random hexamers d(N)-6 (Microsynth, Balgach, Switzerland) as primers. The 16S rRNA, *nrf*A and *nrf*H genes were targeted by RT-PCR and qPCR. For each gene, primers specific to *D. reducens* were used: 16S rRNA, Dred_16S_F (5′-AAA ACG GAG GAA GGT GGG GA-3′) and Dred_16S_R (5′-CTC CTT GCG GTT AGC TCA CC-3′); *nrf*A, nrfA_F(5′-AGA GTT TTA CGA GCC CCG GA-3′) and nrfA_R(5′-AAT GCT GGC CTG CTG ATA CG-3′); *nrf*H, nrfH_F (5′-CAT TAT GGA TCC CTG GGT TG-3′); and nrfH_R (5′-GTC CTG ACC ACG GTC ATT CT-3′). The primers for the 16S rRNA and *nrf*A genes were designed with Primer BLAST, those for the *nrf*H gene were designed by Junier et al. (2010). PCR was carried out using the GoTaq DNA polymerase (Promega, Madison, WI, USA). The KAPA SYBR Fast qPCR kit was used with the Rotor-Gene 3000 thermocycler (Corbett Research, Australia) to perform comparative qPCR. The Rotor-Gene 6 software was used to run the qPCR, perform automatic melting curve analysis and extract Ct values. These were used to calculate the ΔCt (*nrf*A or *nrf*H relative to 16S rRNA) and ΔΔCt (during Fe-reduction relative to fermentation).

### Transmission electron microscopy (TEM)

For the identification of external appendages by TEM, samples were deposited as whole mounts on 400 mesh carbon-coated copper grids (Quantifoil Micro Tools, GmbH Jena). The grids were imaged within 1 h with a Philips/FEI CM12 LaB_6_ microscope at 100 kV onto a Gatan 1024 × 1024 pixel MultiScan CCD camera. Images were recorded and processed using Gatan Digital Micrograph software. On the order of 20 fields of view were observed for fermenting, Fe(III) citrate reducing and HFO reducing cell samples (20, 35, and 25, respectively).

Whole mounts of the products of Fe(III)-citrate and HFO reduction, deposited on carbon-coated gold or copper grids (Quantifoil Micro Tools, GmbH Jena), were imaged with a FEI CM300UT FEG-UT microscope. Selected area electron diffraction (SAED) of micron-scale areas and Fourier Transforms of high-resolution transmission electron microscopy (HR-TEM) images were used for phase identification with the Java Electron Microscopy Software (JEMS) (Stadelmann, 2012); the structural data of iron minerals was taken from the Inorganic Crystal Structure Database (ICSD) (ICSD, FIZ Karlsruhe, Germany and NIST, U.S. Department of Commerce, 2012).

### Protoplast formation and cell shaving

Fermentation and Fe(III)-citrate reducing cultures (400 mL, pellet wet weight ~100 mg) were harvested -after 1 or 2 days incubation, respectively- by centrifugation at 8000 × g for 10 min, and washed in 50 mM TRIS buffer pH 7.1 (Tris-HCl, Acros, Geel, Belgium).

Protoplasts were obtained by re-suspending the cell pellets in 2 mL of an enzymatic mix composed of 1 mg/mL lysozyme (molecular biology grade, Applichem, Damstadt, Germany), 60 μg/mL mutanolysin (from *Streptomyces globisporus*, Sigma) and 50 μg/mL lysostaphin (from *Staphylococcus staphylolytucus*, Sigma) in 0.5 M sucrose and 50 mM TRIS buffer (pH 7.1), amended with 0.48 mg/mL 4-(2-Aminoethyl) benzenesulfonyl fluoride hydrochloride (AEBSF, Applichem). The suspensions were incubated at 37°C and monitored over time by optical microscopy (Eclipse E800, Nikon, Egg, Switzerland) until rod-shaped cells were converted to round bodies by cell wall digestion. After 45 min, the majority (>90%) of the fermentation samples were converted to protoplasts; the iron reduction samples were incubated for 1 h, then half the sample was removed for further processing while the rest was incubated for another hour, before further processing, to increase the protoplast yield (~50%, which did not increase with longer incubations). Protoplasts were harvested by centrifugation at 1000 × g for 10 min (benchtop Eppendorf centrifuge 5415R, Hamburg, Germany). The supernatant was filtered (0.2 μm pore size, PTFE filters, BGB, Geneva, Switzerland) to remove residual cells or protoplasts. Protein concentration was estimated using the Pierce BCA Protein assay (Fisher Scientific, Rockford, IL, USA) and samples were diluted to the same concentration, then stored at −20°C until SDS-PAGE (sodium dodecyl sulfate polyacrylamide gel electrophoresis) was carried out. SDS-PAGE was performed on a 15% acrylamide gel. Prior to loading the samples on the gel, they were mixed 1:1 v/v with the loading buffer (125 mM Tris buffer at pH 6.8, 4% SDS, 20% glycerol, 0.04% bromophenol blue, freshly amended with 5% v/v ß-mercaptoethanol, Applichem) and boiled in a water bath for 15 min. Gels were stained for protein with Coomassie blue. Each lane was excised and digested for liquid chromatography-tandem mass spectrometry (LC-MS/MS) analysis.

Cell shavings were obtained by treating the cells with trypsin agarose (10 units, Sigma) in 0.5 M sucrose and 50 mM TRIS buffer, pH 7.1. As a control, part of the cells was resuspended in the TRIS-sucrose solution without trypsin (shed proteins). Cells were incubated for 30 min at 37°C, with gentle shaking (70 rpm). After incubation, cells were harvested by centrifugation and the supernatant was collected. 1 mM dithiothreitol (DTE molecular biology grade, LubioScience, Luzern, Switzerland) and 1 mM iodoacetamide (Applichem) were added sequentially to the supernatant. As above, samples were diluted to the same concentration prior to storage at −20°C for LC-MS/MS analysis.

**Figure 1:**
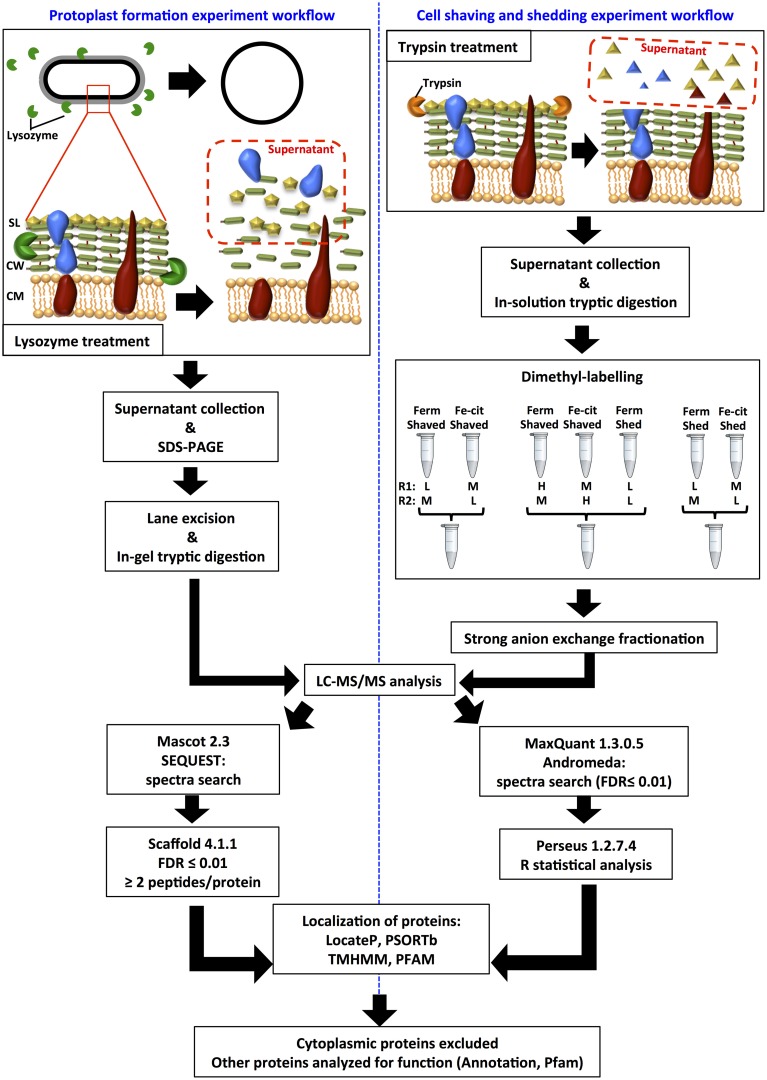
**Schematic representation of the workflow strategy for the protoplast formation and cell shaving experiments**. In the protoplast formation, lysostaphin and mutanolysin were also used, as well as lysozyme. In the cell shaving, agarose-bound trypsin was used, and a control without enzyme (cell shedding) was included. In both experiments, cells were collected during fermentation (Ferm) and during Fe(III)-citrate reduction with lactate (Fe-cit). Shaved or shed samples were labeled with light (L), medium (M), or heavy (H) dimethyl reactants, and mixed in 1:1(:1) ratios in different combinations, as depicted. Each mix was prepared in duplicate, with inverted labeling (R1 and R2).

All experiments were performed in biological duplicates. Figure 1 depicts a schematic of the workflow for the two surface-protein extraction experiments.

### LC-MS/MS analysis of cell wall extracts (protoplast formation)

Entire lanes of SDS-PAGE gels were sliced into pieces. Samples were first washed twice for 20 min in 50% ethanol, 50 mM ammonium bicarbonate (AB). Gels slices were dried down by vacuum centrifugation. All samples were reduced/alkylated using DTE and iodoacetamide. Gel pieces were dried again and rehydrated using a trypsin solution (12.5 ng/μl in 50 mM AB and 10 mM CaCl_2_). Trypsin digestion was performed overnight and the resulting peptides were extracted twice for 20 min in 70% ethanol and 5% formic acid (FA). Samples were dried down and resuspended in 2% acetonitrile and 0.1% FA for LC-MS/MS injections. One-dimensional liquid chromatography separation was performed using a Dionex Ultimate 3000 RSLC nano UPLC system (Dionex) on-line connected with an Orbitrap Q Exactive Mass-Spectrometer (Thermo Fischer Scientific). A custom made capillary pre-column (Magic AQ C18; 3 μm–200 Å; 2 cm × 100 μm ID) was used for sample trapping and cleaning. Analytical separation was then performed using a C18 capillary column (Nikkyo Technos Co; Magic AQ C18; 3 μm-100 Å; 15 cm × 75 μm ID) at 250 nl/min. using the following mobile phases: A (98% acetonitrile, 0.1% FA) and B (90% acetonitrile, 0.1% FA). Separation of peptides was carried over an 85 min biphasic gradient (51 min. up to 30% B and then 62 min. up to 47% B). Mass spectrometric measurements were performed using a data-dependent top 20 method, with the full-MS scans acquired at 70 K resolution (at m/z 200) and MS/MS scans acquired at 17.5 K resolution (at m/z 200).

LC-MS/MS spectra were searched against the predicted *D. reducens* proteome obtained from the National Center for Biotechnology Information (NCBI) (Geer et al., 2010) using Mascot 2.3 (Matrix Science) and SEQUEST in Proteome Discoverer v.1.3. All searches were performed with Trypsin cleavage specificity, up to 2 missed cleavages allowed and ion mass tolerance of 10 ppm for the precursor and 0.05 Da for the fragments. Carbamidomethylation of cysteines was set as a fixed modification, whereas oxidation (M), acetylation (Protein N-term), phosphorylation (STY) were considered as variable modifications. Scaffold 4.1.1 (Proteome Software) was used for further data analysis. Results were filtered with a protein threshold FDR of 0.01, and a minimum of two peptides per protein. The spectrum counting label-free quantitative method of Scaffold (normalized total spectra) was used to compare different conditions: the sum of the total spectrum counts for all proteins identified within each sample is adjusted to a common value (i.e., the average of the sums of all the samples) by applying a scaling factor to each protein. This normalization method is valid and allows for comparison of protein counts when the total protein loaded is comparable amongst samples, such as in our case (Scaffold User Manual). The spectrum counts for each protein were averaged for the two fermentation duplicates and for the Fe(III) reduction samples (part of the two duplicates treated for 60 min, the rest treated for 120 min). A protein was considered to be more expressed in one condition relative to the other if the spectrum count was at least twice as high in this condition, and higher by an absolute value of at least five (to avoid the unreliability of very low counts), also accounting for the error (standard deviation for the Fe(III) reducing samples, semi-difference for the fermentation samples).

### Dimethyl labeling and SAX fractionation of shaved and shed proteins

Each sample (10 μg) was reconstituted in 50 μl of 4 M Urea, 10% acetonitrile and buffered with Tris-HCl pH 8.5 to a final concentration of 30 mM. Proteins were reduced in 10 mM DTE at 37°C for 60 min. Cooled samples were subsequently incubated in 40 mM iodoacetamide at 37°C for 45 min in a light-protected environment. Reaction was quenched by addition of DTE to a final concentration of 10 mM. A first digestion step was then performed using Lys-C (1:50 enzyme: protein) for 2 h at 37°C. After diluting the samples 5-fold, a second digestion step was performed overnight at 37°C using Mass Spectrometry grade trypsin gold (1:50 enzyme: protein) and 10 mM CaCl_2_. The reaction was stopped by addition of 2 μl of pure FA and peptides were concentrated by vacuum centrifugation to a final volume of 70 μl. Samples were dimethyl-labeled as previously described (Boersema et al., 2009). Three experiments were conducted. In the first experiment, the shed-proteins from fermentation cells sample were labeled with light dimethyl reactants (CH_2_O + NaBH_3_CN), the shed-proteins from Fe(III)-reducing cells sample was labeled with medium reactants (CD_2_O + NaBH_3_CN); duplicate samples were labeled in opposite ways. In the second experiment the shaved-proteins from the fermentation sample were labeled with light dimethyl reactants, the shaved-proteins from the Fe(III)-reducing sample were labeled with medium reactants; duplicate samples were labeled in opposite ways. In the last experiment, the proteins shed from the fermentation sample were labeled with light dimethyl reactants, the shaved proteins from the fermentation sample were labeled with medium reactants, and the shaved proteins from the Fe(III)-reducing sample were labeled with heavy methyl reactants (13CD_2_O + NABH_3_CN); the labeling was swapped for the duplicates of the latter two samples. As a final step of the labeling procedure, samples were mixed in 1:1:1 (vol:vol:vol- Light: Medium: Heavy) ratio and lyophilized. Strong anion exchange (SAX) fractionation was performed as previously described (Wiśniewski et al., 2009). The eluted fractions were dried by vacuum centrifugation and used for LC-MS analysis.

The mass spectrometry proteomics data were deposited to the Proteome × change Consortium (Vizcaino et al., 2013) via the PRIDE partner repository with the dataset identifier PXD001072 and DOI 10.6019/PXD001072.

### Mass spectrometry and data analysis of dimethyl labeled peptide samples

Each SAX fraction was resuspended in 2% acetonitrile, 0.1% FA for LC-MS/MS injections and then loaded on the same pre-column and separated on the same C18 tip-capillary column as for the protoplast formation samples. MS/MS data was acquired in data-dependent mode (over a 4 h acetonitrile 2–42% gradient) on an Orbitrap Q exactive Mass spectrometer equipped with a Dionex Ultimate 3000 RSLC nano UPLC system and custom made nanoESI source. Acquired RAW files were processed using MaxQuant version 1.3.0.5 (Cox et al., 2009) and its internal search engine Andromeda (Cox et al., 2011). MS/MS spectra were searched against the predicted *D. reducens* proteome. MaxQuant default identification settings were used in combination with dimethyl-labeling parameters. Search results were filtered with a false-discovery rate (FDR) of 0.01. Known contaminants and reverse hits were removed before statistical analysis. Relative quantification between different conditions was obtained by calculating the significance *B*-values for each of the identified proteins using Perseus (Table SI-1) (Cox et al., 2009).

### Data analysis

Proteins identified by LC-MS/MS were screened to remove any cytoplasmic proteins present due to inevitable cell lysis. In addition, the tools used to select the surfaceome proteins were also used to assign, where possible, an identity and a localization to the cell-binding domain for each protein. We used TMHMM (Krogh et al., 2001) to identify hydrophobic domains that could represent membrane spanning domains if multiple domains are present, or either a membrane anchor or a signal peptide, if a single domain is detected. PSORTb (Yu et al., 2011) was used to classify the proteins according to the following cell compartments: cytoplasm (C), cytoplasmic membrane (CM), cell wall (CW), or extracellular. In some cases, the pipeline was unable to assign a precise localization (unknown), but sometimes was able to exclude a cytoplasmic localization. LocateP (Zhou et al., 2008) was used similarly. This tool is able to assign proteins to the same compartments as PSORTb, but in addition provides information of the type of membrane binding (including lipoproteins) and on the secretion mechanism of surface proteins. When possible, we trusted the assignment supported by at least two of the tools. In cases of conflicting assignment, we used information available on the protein and its function to make a putative assignment. The Pfam database (Punta et al., 2011) was used to identify conserved domains in all the surface proteins to obtain information on their function and to identify CW binding domains: CWBD_1 (PF01473), CWBD_2 (PF04122), GW-domain (PF13457), LysM-type domain (PF01476), SLHD (PF0039). LPxTG-domains were identified by LocateP.

## Results

### Fe(III) reduction with lactate as an electron donor

**Figure 2:**
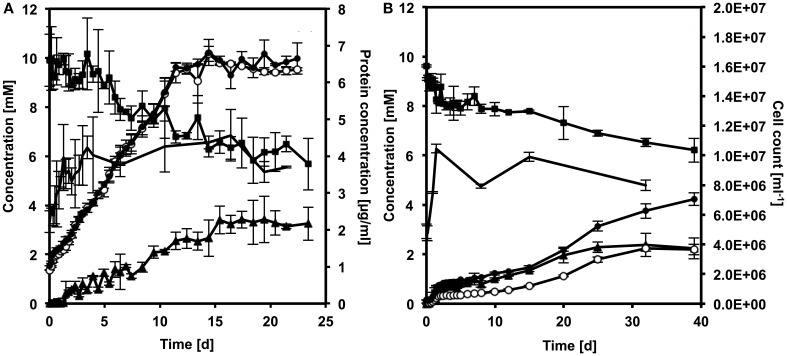
**Fe(III)-citrate (A) and HFO (B) reduction plots with lactate as the electron donor**. The curves represent concentrations over time of: total extractable Fe(II) (full circles), soluble Fe(II) (empty circles), electron donor (squares), acetate (triangles), protein content [no marker, in **(A)**] or cell count [no marker in **(B)**]. Error bars represent standard deviation from triplicate samples.

*D. reducens* is capable of reducing both soluble and insoluble Fe(III), in the forms of Fe(III)-citrate and the environmentally relevant HFO, respectively. Figure 2 shows increasing concentrations of soluble and extractable Fe(II) derived from the reduction of Fe(III)-citrate (Figure 2A) and HFO (Figure 2B). Additionally, electron donor consumption, buildup of acetate from electron donor oxidation, and protein accumulation were also quantified. Killed, lysed and no-cell controls are unable to reduce Fe(III) (Figure SI-1).

Fe(III)-citrate reduction is complete, and yields mainly soluble Fe(II), as is apparent from the overlap of the soluble and extractable Fe(II) curves in Figure 2A. However, small amounts of dark precipitate formed in the culture. TEM observations and X-ray energy dispersive spectroscopy (EDS) analysis confirmed the presence of Fe-containing precipitates (Figure SI-2A). It is likely that the precipitate passes through the filter and is measured as soluble Fe(II). HFO reduction, instead, is incomplete and, after 40 days, Fe(II) concentration reaches a plateau, despite the presence of excess electron donor. Over time, the iron solid phase changed from red to dark brown-black. TEM investigations and EDS and SAED analysis revealed the product of HFO reduction to be magnetite (Fe_3_O_4_), a mixed Fe(II)-Fe(III) mineral phase (Figure SI-2B-D).

Reduction of both Fe(III) species is concomitant with lactate oxidation to acetate. However, *D. reducens* does not seem capable of significant energy conservation from this process: a small amount of growth was detected, but only in the first 2 days of Fe(III) reduction and thus growth cannot be temporally correlated to Fe reduction. Furthermore, more lactate is consumed than is necessary for the amount of reduced iron (the predicted stoichiometry of lactate oxidized: Fe(III) reduced is 1: 4).

### *D. reducens* requires direct cell surface contact to reduce HFO

**Figure 3:**
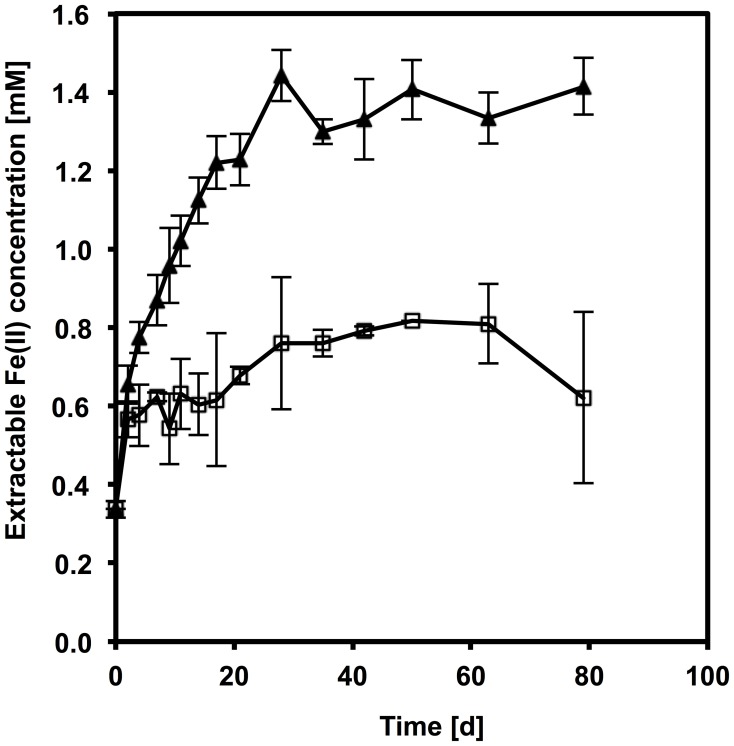
**Available (full triangles) and unavailable (glass-HFO) (empty squares) HFO reduction by *D. reducens* with lactate as an electron donor**. Error bars represent standard deviation from triplicate samples.

When *D. reducens* was incubated with glass-HFO and lactate only 0.5 mM Fe(II) accumulated in the medium, likely accounted for by the reduction of HFO present on the outer surface of the glass particles and thus readily accessible to cells. In the control culture containing HFO not embedded in glass particles, 1.5 mM Fe(II) were measured (Figure 3). This suggests that *D. reducens* cells require direct contact with the solid electron acceptor to reduce it and do not employ a soluble electron shuttle. The absence of a soluble reducing compound was supported by a test of the AQDS reducing capability of the spent medium. The culture medium was filter-sterilized at inoculation time (i.e., prior to HFO reduction) and once HFO reduction was underway, and was amended with 1 mM AQDS. After 48 h, measurement of residual AQDS revealed that none had been reduced to AH_2_DS, consistently with the absence of a soluble reduced compound in the medium.

**Figure 4:**
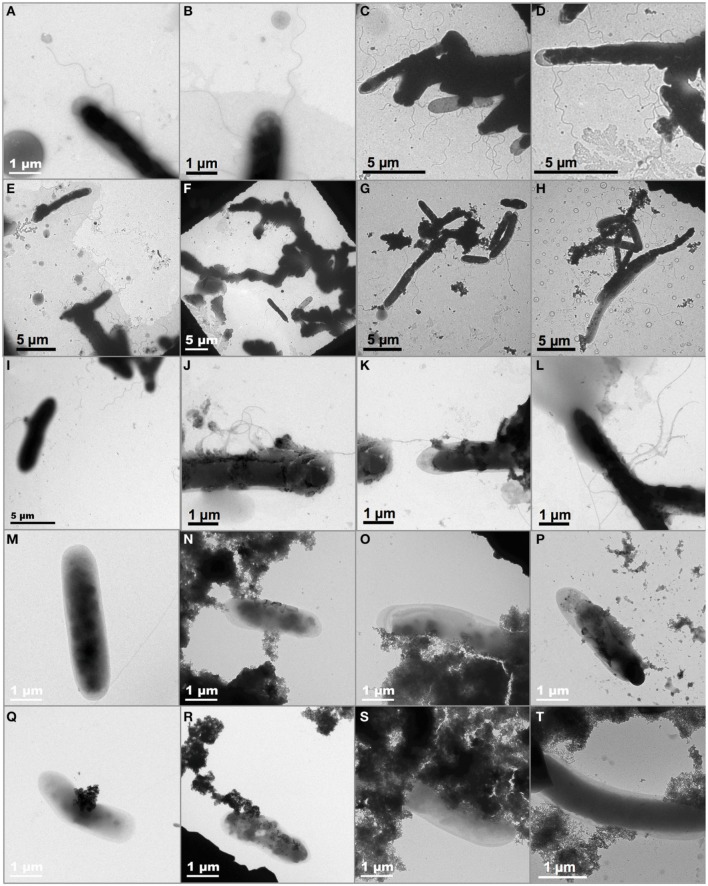
**TEM micrographs investigating the presence of external appendages (indicated by arrows) protruding from *D. reducens* cells under different conditions: fermentation (A–F), Fe(III)-citrate reduction with lactate (G–L) or HFO reduction with lactate (M–T) as an electron donor**. Appendages were observed in all conditions except for HFO reduction with lactate.

The possibility that *D. reducens* may use external appendages as conductive “nanowires” to transfer electrons from the cell to the external TEA, HFO, was tested by TEM observation of whole-mount samples prepared under different conditions. Cultures that were fermenting pyruvate or reducing Fe(III)-citrate or HFO with lactate as an electron donor were interrogated (Figure 4). Our observations showed evidence for the presence of external appendages extending from cells grown in the presence of pyruvate (20 fields of view observed) and in the sample with Fe(III)-citrate and lactate (35 fields of view observed), although from TEM observation alone, it is difficult to assess the nature of the appendages, so they could be either pili or flagella. No evidence could be found for their presence in any of the 25 fields of view observed containing cells from the sample with HFO and lactate, also suggesting the requirement for direct contact between the cell surface and the external TEA. In contrast, appendages were readily identifiable in cultures grown with pyruvate and HFO, suggesting that the presence of HFO does not obscure their observation.

### *c*-type cytochromes are not likely to be involved in Fe(III) reduction

*c*-type cytochromes are the most obvious candidate enzymes for Fe(III) reduction. There are two enzymes in the genome of *D. reducens* that are predicted to be *c*-type cytochromes. These proteins are encoded for by the *nrf*H and *nrf*A genes, both containing heme-binding domains (CxxCH), and are predicted to be tetraheme cytochromes. Therefore, we probed the involvement of these two proteins in Fe(III) reduction. Qualitative observations derived from RT-PCR and comparative quantitative expression data derived from qRT-PCR, both carried out with *D. reducens*-specific primers for 16S rRNA, *nrf*H, and *nrf*A, provide no evidence for the involvement of NrfH and NrfA in Fe(III) reduction with lactate as an electron donor.

The RT-PCR electrophoresis gel displayed no visible band for *nrf*H and a very weak band for *nrf*A in the Fe(III)-citrate reduction sample, in contrast to the significantly more obvious bands visible for both genes in pyruvate fermentation conditions. Consistently, the expression levels for *nrf*H and *nrf*A relative to the 16S rRNA gene (2^∧^(−ΔCt)) were found to be two orders of magnitude lower during Fe(III)-citrate reduction than pyruvate fermentation (2^∧^(−ΔΔCt)) (Table SI-2 and Figure SI-3).

### The surfaceome of *d. reducens*

We used two methods (mentioned in Solis and Cordwell, 2011) to extract surface exposed proteins from *D. reducens* cells incubated either with pyruvate or with Fe(III)-citrate and lactate. The first is cell shaving: cells were exposed to agarose-bound trypsin in order to cleave peptide fragments protruding out of the cell surface; we included a shed protein control with cells incubated in the absence of trypsin. The second method consisted of enzymatically hydrolyzing the cell wall in order to release cell wall proteins and loosely bound outward-facing membrane proteins. The proteins extracted were trypsin digested and identified by LC-MS/MS analysis and subsequently screened for the presence of cytoplasmic proteins. We found that neither method was completely successful in enriching for solely surface exposed proteins: a total of 599 proteins were extracted in the protoplast experiment, and 767 were initially identified by the MaxQuant software in the shaving experiment, but this dataset was reduced to 469 by a 1% FDR filtration with Scaffold and used for subsequent in silico analysis. Even though this indicates cell lysis, it nonetheless represents a small subset of the total proteome of *D. reducens* (Figure SI-4). We used cell localization algorithms (mainly TMHMM, LocateP and PSORTb) to identify which proteins pertain to the cytoplasm and excluded them from the analysis that followed, which concerns only the putative surface proteins. The complete datasets before cytoplasmic-protein removal can be found in Tables SI-3 and SI-4, and Table 1 summarizes the number of proteins identified in each experiment and pertaining to the cytoplasm or the surfaceome. In addition, all raw proteomic data was uploaded to the PRIDE database with under the entry PXD001072 (DOI 10.6019/PXD001072).

**Table 1:** **Number of proteins identified in the protoplast (P) and shaving/shedding (Sh) experiments**.

		**Total extract**	**Surface**	**Cytoplasm**
Total	P	599	111	488
	Sh	469	79	390
Unique	P	238	47	191
	Sh	108	15	93
Shared	P&Sh	361	64	297

Our investigation aims at characterizing the surfaceome of *D. reducens*, focusing in particular on the identification of surface enzymes with redox activity, potentially involved in electron transfer to extracellular TEAs, such as Fe(III)-citrate and HFO. The purpose of comparing Fe(III)-citrate reducing and pyruvate fermenting cells is to identify significant expression differences which may provide additional insight into the specific involvement of proteins during Fe(III) reduction.

Overall, few identified proteins were found to be differentially expressed: (i) those for which the semi-quantitative spectrum count in the protoplast experiment exhibited a value at least double in one condition relative to the other or (ii) those for which the comparative analysis of shaved/shed proteins indicated a statistically significant difference in expression level. The entries are highlighted in Table SI-5. Probably due to the disturbance resulting from cell lysis, it was difficult to detect significant differences between the shaved and the shed proteins, and most surface proteins identified in the shaving experiment were also present in the shed cell control. Significance *B*-values and relative quantifications between different conditions in the shaving/shedding experiment is shown in Table SI-1.

In total, we identified 126 putative surface proteins: 64 were present in the extracts from both types of experiments, 15 proteins were identified only in the cell-shaving/shedding experiments and 47 only in the protoplast experiments (Table 2). We attempted to identify the general function as well as the surface-binding mechanism for these proteins, in order to assign a more precise localization to each of them. In the following paragraphs, we describe the functional typologies of proteins we identified in the surface layers of the cell, as well as their specific localization.

**Table 2:** **Protein count according to function, cellular localization [surface-unknown (S-U), cytoplasmic membrane (CM), cell wall (CW), extracellular (EC)], experiment in which they were identified [only protolast (P), only shaving (Sh), both (P&Sh)], differential expression [up-regulated during fermentation (>Ferm) or Fe(III) reduction (>Fe)]**.

	**Protein count**	**Cellular localization**				**Experiment**			**Differential expression**	
		**S-U**	**CM**	**CW**	**EC**	**only P**	**only Sh**	**P&Sh**	**>Ferm**	**>Fe**
Transport	42	4	38	0	0	18	1	23	6	3
Chemotaxis	10	2	6	0	2	2	4	4	1	0
Proteases and CW hydrolases	26	8	14	4	0	14	2	10	4	0
Other or unknown functions	42	2	32	8	0	11	8	23	8	1
Redox	6	0	6	0	0	2	0	4	1	1
Total	126	16	90	10	2	47	15	64	20	5

### Redox-active proteins present in the surfaceome of *d. reducens*

Six surface proteins with predicted redox activity were identified (Table 2 and Table SI-5).

A protein containing a 4Fe-4S cluster binding domain, Dred_0462, is predicted to have one TMH, located close to the C-terminus. Consistently, PSORTb gives it a membrane location. Dred_0462 is a component of a trimeric membrane-bound hydrogenase (Dred_0461–3). Additionally, another subunit of the same protein, Dred_0461, is detected only in the protoplast experiments and is predicted to be a membrane-spanning protein. Finally, the cytoplasmic subunit of the protein (Dred_0463) was also detected among the cytoplasmic proteins: while they were not the focus of this study, we deemed it interesting to evaluate the presence of the third component of this hydrogenase. Dred_0461 was only identified in extracts of the fermentation culture; Dred_0462–3 were identified in the extracts from both experiments under both culturing conditions. However, the protoplast experiment data indicate that both, and in particular the cytoplasmic subunit of the hydrogenase, appear to be up-regulated during fermentation (Table SI-5).

Another 4Fe-4S cluster binding domain-bearing protein annotated as a ferredoxin (Dred_0143) is also predicted to contain one TMH, although at the N-terminus, and to be outward facing, based on TMHMM analysis. However, this topology is not supported by LocateP analysis, which indicates Dred_0143 to be a cytoplasmic protein, nor by PSORTb, which is unable to localize it. Based on this information we are unable to assign with certainty a localization to this protein, but since it is potentially membrane bound, we included it in our list of putative surface proteins. This protein does not appear to be differentially expressed under the two conditions considered.

Two very similar proteins annotated as nitrate reductase gamma subunit were also identified either in the extracts from both experiments (Dred_1445) or in the extract from the protoplast formation experiment (Dred_3199). These two proteins share significant homology (Figure SI-5) and are characterized by 5 membrane spanning domains, suggesting membrane localization. The protoplast experiment data indicate that Dred_1445 may be slightly up-regulated during fermentation relative to Fe(III) reduction; in contrast, neither experiment suggests differential expression for Dred_3199 under the different culturing conditions.

Finally, a protein predicted to be N-terminally anchored through one transmembrane domain and annotated as alkyl hydroperoxide reductase (AhpC type protein, Dred_1533) was identified. Higher peptide counts were measured for this protein in the protoplast formation extracts in the Fe(III) reducing sample relative to fermentation.

### Other functions identified

The majority (~75%) of the surface proteins identified are localized to the membrane (Table 2 and Table SI-5). These proteins are either anchored to the membrane through a single TMH or through a lipid anchor (lipoproteins), or they have multiple TMHs that span the membrane. A few are cell wall-bound, through a LysM-type domain (PF01476), or through a SLHD (PF00395). We were unable to identify any other cell-wall binding domain [i.e., CWBD_1 (PF01473), CWBD_2 (PF04122), LPxTG or GW domain (PF13457)] in any of the surface proteins. For a few proteins (16) we were unable to identify a precise localization, although they are almost certainly surface proteins since they are predicted to be secreted proteins.

**Figure 5:**
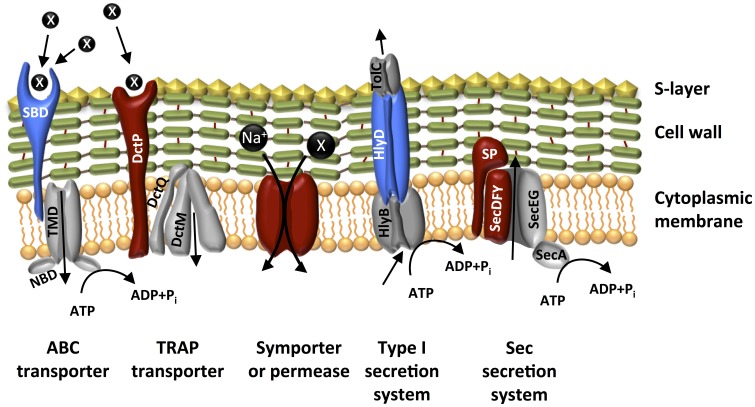
**Model of the localization of surface proteins involved in transport in *D. reducens***. Gray coloring indicates proteins expected to belong to the transport complex, but that were not identified in our extracts, while colored proteins are those identified: blue coloring indicates lipoproteins, red indicates membrane spanning proteins; black circles represent solutes (unspecified, if marked with an X). SBP, solute binding protein; NBD, nucleotide binding domain; SP, signal peptidase.

Unsurprisingly, a significant fraction of the surface proteins are involved in transport (Table 2 and Table SI-5). Particularly dominant are the proteins responsible for substrate binding, although the specificity of their substrate is unknown in most cases. A few of these receptors are predicted to bind to specific compounds (e.g., amino acids, phosphate). Consistent with their function, all the solute binding proteins are predicted to be lipid anchored to the membrane, but localized extracellularly. The protoplast experiment data indicates three substrate binding proteins to be up-regulated during Fe(III)-reduction, but their substrate specificity is unknown. Most of the identified transport-related proteins belong to ABC-type (ATP binding cassette) transporters, characterized by the use of ATP to fuel substrate transport (Van der Heide and Poolman, 2002). However, a few tripartite ATP-independent periplasmic (TRAP) type transporters were also identified (Dred_0407 and Dred_2757). These transporters obtain energy to actively channel substrates from the extracellular environment to the cytoplasm by combining it with the thermodynamically favorable transport of a solute such as Na^+^ (Mulligan et al., 2011). A proton-translocating pyrophosphatase, predicted to be up-regulated during fermentation (according to the protoplast experiment), was also identified. Figure 5 depicts a model of transport-related proteins in *D. reducens*.

The other transport-related proteins identified in both extracts are involved in secretion. Among these, a few (Dred_3060, 3141, 1670, and 0235) seem to be up-regulated during fermentation, according to the protoplast experiment data.

Eighteen transport-related proteins were identified in the protoplast protein extract only. Many of these are the transmembrane region of transporters, symporters, or permeases, involved in the import of specific substrates such as glycerol, lactate, magnesium, and uracil. They are characterized by multiple TMHs. A few proteins extracted in the protoplast experiment are involved in solute binding or in secretion. Two of the identified proteins in this dataset are annotated as glycine/betaine ABC transporters (Dred_3207, Dred_0473). These transporters are involved in the regulation of osmolarity stress (Van der Heide and Poolman, 2002).

**Figure 6:**
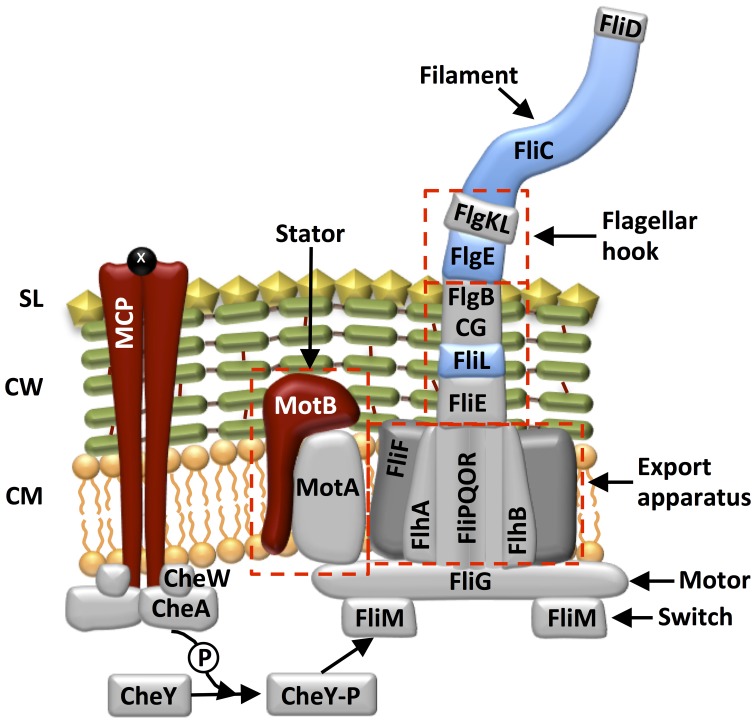
**Model of the localization of surface proteins involved in chemotaxis in *D. reducens***. Gray coloring indicates proteins that are expected to be involved in chemotaxis, but were not identified in our extracts, while colored proteins are those extracted: blue coloring indicates extracellular proteins, red indicates membrane spanning proteins. SL, S-layer; CW, Cell wall; CM, cytoplasmic membrane; MCP, methyl-accepting chemotaxis protein. This model is based on the flagellum model depicted in Desvaux et al. (2006) and the chemotaxis model described by Wadhams and Armitage (2004).

Several proteins related to chemotaxis were also identified: methyl-acceptor chemotaxis proteins, flagella components and few putative type IV pili subunits (Table 2 and Table SI-5, Figure 6).

Another significant functional class identified among the surface proteins consists of proteases and cell wall hydrolases. Four among the proteases and CW-hydrolyzing proteins are up-regulated during fermentation. In addition to the PG hydrolases, two proteins potentially involved in CW synthesis were identified: Dred_1646 and Dred_0669, both of which contain penicillin-binding domains.

In the protoplast-formation extract a couple of other proteins involved in cell growth were identified: FtsQ (Dred_0679), a protein predicted to be involved in cell division, and MreC (Dred_2547) predicted to be involved in rod shape determination.

### Proteins of unknown function

Several other proteins were identified in the cell surface extracts. Most of the membrane bound proteins (mainly lipoproteins) contain domains whose function is unknown, thus it is not possible to hypothesize about their role. Some, however, have domains that could be involved in protein maturation, suggesting that they are involved in protein folding or multi-complex formation after transfer across the membrane. Other putative functions derived by the presence of certain domains in surface proteins include the modulation of the activity of ion channels (PF02950), substrate binding and/or possibly signaling (PF03180 and PF12262), and adhesion. The homolog of *B. subtilis* SpoIIIAH sporulation protein (Dred_1063) was also identified in the protein extracts. This is a mother cell membrane protein involved in channeling nutrients or signal molecules from the mother cell to the forespore during sporulation (Higgins and Dworkin, 2012). Since sporulation was not ongoing during protein extraction, it is possible that this protein plays also a role in vegetative cells. If this were the case, presumably this role would be similar to that played during spore formation, such as substrate or protein export across the membrane.

Some proteins identified are predicted to be cell wall-bound. A LysM-type protein contains a domain related to cell division, and in particular to formation of the division septum (PF04977). Others are S-layer proteins with either unknown functions or putative functions such as proteolysis or adhesion (PF14620, PF03413, PF13620).

## Discussion

*D. reducens* is known to be capable of metal reduction (Tebo and Obraztsova, 1998). We previously investigated the mechanism of Fe(III) reduction with pyruvate, a fermentable substrate, as the electron donor (Dalla Vecchia et al., 2014). Here, we have shown that, in the presence of lactate, a non-fermentable substrate, *D. reducens* is capable of reducing HFO, a poorly-crystalline Fe(III)-oxide, in addition to its previously shown ability to reduce soluble Fe(III), in the form of Fe(III)-citrate (Tebo and Obraztsova, 1998).

Lactate consumption and the resulting accumulation of acetate in the growth medium occur concomitantly with the reduction of Fe(III) suggesting that these processes are directly linked, although more lactate is consumed than what is stoichiometrically required to reduce Fe(III) to the measured amount of Fe(II). This suggests that some electrons may be stored within the cells when they oxidize lactate, as was previously observed with pyruvate as the electron donor (Dalla Vecchia et al., 2014). Very limited, though detectable, growth was measured during iron reduction, and the most significant increase in cell concentration occurred at the onset of reduction. This suggests that *D. reducens* is able to conserve only a small fraction of the energy associated with Fe(III) reduction when coupled to lactate oxidation. Our hypothesis is that under these conditions, *D. reducens* is capable of conserving the energy required for cell maintenance, but not for significant growth. This is in contrast to the metabolism fueled by pyruvate. This substrate is rapidly consumed and supports growth, while Fe(III) is reduced more slowly, and acts as a fortuitous electron sink, rather than as a TEA for respiratory growth (Dalla Vecchia et al., 2014). This is not the only difference between Fe(III) reduction with the two electron donors: we found that the mechanism harnessed by *D. reducens* cells to transfer electrons to Fe(III), in particular to the solid phase, is significantly distinct when lactate is present, relative to pyruvate.

During pyruvate fermentation, electrons are conveyed to the extracellular electron acceptor, i.e., HFO, by means of a soluble electron carrier, riboflavin, which is shuttled between the TEA and the cell (Dalla Vecchia et al., 2014). In the presence of lactate, conversely, we found that spent medium from HFO-reducing cultures did not reduce AQDS. This suggests the absence of a reduced soluble compound. This finding is bolstered by the fact that *D. reducens* is unable to reduce physically inaccessible glass-HFO with lactate as an electron donor (Figure 3), which indicates that this microorganism requires direct contact with the solid TEA. Direct physical contact between cells and solid phase extracellular Fe(III) is a widely documented mechanism employed for electron transfer among Gram-negative metal-reducing bacteria, and has been characterized in great detail in two model organisms, *Geobacter sulfurreducens* PCA and *Shewanella oneidensis* MR-1 (Weber et al., 2006, and references within). In these microorganisms, for electron transfer by direct contact, electrons are transported from the quinone pool in the cytoplasmic membrane across the periplasm through a chain of multi-heme *c*-type cytochromes, which culminates with an outer membrane embedded, outward-facing *c*-type cytochrome (Schroder et al., 2003; Shi et al., 2007). At this point, electron transfer from this cytochrome to the TEA can occur either by direct physical contact (Lower et al., 2005; Gorby et al., 2006; Gralnick and Newman, 2007; Shi et al., 2009; Inoue et al., 2011), or can be mediated by electron conductive pili (Reguera et al., 2005; Gorby et al., 2006). These mechanisms cannot be conserved in Gram-positive bacteria, due to the different cell morphology characterized by a single membrane and a thick cell wall, which, typically, is not expected to contain proteins involved in electron transfer. Carlson et al. (2012) found *c*-type cytochromes loosely bound to the outer surface of the CW of the Gram-positive bacterium *Thermincola potens*, and suggested that these could be involved in solid-phase Fe(III) reduction. However, here we show that, in the presence of lactate, as was shown also in the presence of pyruvate, the two *c*-type cytochromes encoded for in the genome of *D. reducens*, NrfA, and NrfH, do not appear to be up-regulated during Fe(III) reduction (Figure SI-3, Table SI-2). This result is supported by the fact that no peptides belonging to these proteins were identified by LC-MS/MS in the surfaceome of *D. reducens* under any condition (Tables SI-3–SI-5). Also, pili do not appear to be expressed as a response to the presence of an insoluble TEA in *D. reducens*: TEM observations evidenced the presence of external appendages, potentially pili, extending from the surface of cells grown in the presence of pyruvate, and of Fe(III)-citrate and lactate, but not in the presence of HFO and lactate (Figure 4). Thus, nanowires are unlikely to represent a major route for extracellular electron transport in this organism.

Given these observations, it appears that no previously existing model for bacterially mediated metal-reduction is applicable to Fe(III) reduction with lactate as electron donor by *D. reducens*: despite the requirement for direct contact with the TEA, *c*-type cytochromes do not appear to be involved. The absence of detectable levels of *c*-type cytochromes during metal reduction was also reported in *Pelobacter carbinolicus* and *Desulfitobacterium metallireducens*, but no putative mechanism for the reaction was proposed (Lovley et al., 1995; Finneran et al., 2002). The most likely scenario is that electrons are transferred from the quinone pool in the membrane and across the CW through a (or multiple) surface protein(s) with an outward-facing domain, capable of reducing extracellular TEAs through direct contact.

**Figure 7:**
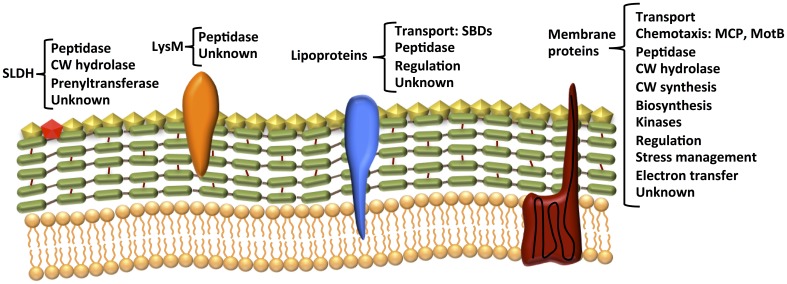
**Our data indicate that the surfaceome comprises proteins bound to the surface through SLDH (bright red), LysM-like domains (orange), anchors to the membrane lipids (blue) and TMHs (dark red)**. Different localizations and binding types are associated to different general functions, which we list in this figure.

To probe this possibility, we investigated the surfaceome of *D. reducens* during pyruvate fermentation and during Fe(III)-citrate reduction with lactate. We found that this cell compartment exhibits a variety of functions, the great majority of which are not differentially expressed in the two incubation conditions (Figure 7, Table 2 and Table SI-5). The most dominant function is related to transport: either the import of extracellular substrates toward the cytoplasm (through ABC transporters, TRAP systems, symporters or permeases), or the export of surface proteins toward their final localization (Figure 5). Other surface proteins identified are (i) signal recognition or transducer proteins, (ii) proteases, or (iii) redox-active proteins.

The six redox-active proteins identified were considered for their possible involvement in Fe(III) reduction. These proteins are listed in Table SI-5. Based on the information available about these proteins or their homologs in other species, Dred_1445 and Dred_3199 are highly unlikely to be involved in Fe(III) reduction.

A surface protein which could be involved in Fe(III) reduction is Dred_0462. This protein, annotated as a ferredoxin, is actually a component of a trimeric hydrogenase (Dred_0461–3), all subunits of which were identified in our extracts, although Dred_0461 was only identified in the fermentation sample. Dred_0461 is a putative cytochrome *b* subunit, predicted to have 10 TMH, while Dred_0463 is the iron containing catalytic unit of the complex, and contains no TMH, thus it is predicted to be cytoplasmic. Its presence in our extracts is likely due to cell lysis and cytoplasmic contamination. This trimeric hydrogenase has been proposed to be responsible for the oxidation of H_2_ during U(VI) reduction by *D. reducens* (Junier et al., 2010). By analogy, we initially considered it a candidate for involvement in Fe(III) reduction. However, in the cultures we used, H_2_ is not the intended electron donor. The fermentation culture releases H_2_ (Dalla Vecchia et al., 2014) and the Fe(III)-citrate reducing culture is not expected to include H_2_ since the electron donor is lactate, unless minor intracellular concentrations of H_2_ are carried-over at inoculation time. Furthermore, Dred_0463, and possibly also Dred_0462, seems to be down-regulated during Fe(III) reduction. This suggests that the trimeric hydrogenase might be functional only under fermentative conditions. At this point it cannot be excluded, however, that Dred_0462 alone may still be active as a ferredoxin also during Fe(III) reduction, and could be directly involved in this process.

Another putative surface protein, also annotated as a ferredoxin, which could be involved in Fe(III) reduction, is Dred_0143. Its localization to the surface is not certain and will require future validation. In addition to the FeS-binding domain, Dred_0143 also includes an NADH binding region, and belongs to the pyridine nucleotide-disulphide oxidoreductase family of proteins (PF07992). Moreover, this protein has two heterodisulfide reductase subunit A domains, suggesting this protein is a heterodisufide reductase (Hdr) (Junier et al., 2010). Its genomic locus is in a region that comprises two repeats, including other genes related to electron transfer [FeS proteins, oxidoreductases and the delta subunit of methyl viologen reducing hydrogenase (*mvh*)].

The co-localization of *hdr*s and of *mvh*s is typical of methanogenic archaea, which are capable of bifurcating electrons derived from H_2_ oxidation to associate the exoergonic reduction of CoM-S-S-CoB, which yields CoB-SH and CoM-SH, to the endergonic reduction of ferredoxin (Fd). This process is catalyzed by a protein complex that includes three Hdr subunits (A, B, and C) and a hydrogenase (Thauer et al., 2008; Pereira, 2011). HdrA is hypothesized to be the Fd reductase (Costa et al., 2010). In methanogens, these protein complexes are soluble, and the absence of a transmembrane component hinders the possibility to satisfy chemiosmotically the energetic requirement for Fd reduction, hence the use of electron bifurcation. In sulfate-reducing bacteria Hdr-type proteins are common; however, the role they play is not well defined. It has been suggested that they may be intermediate electron carriers in the sulfate reduction pathway (Strittmatter et al., 2009; Junier et al., 2010), although in most instances not all the components of the complex (HdrABC and a hydrogenase) are encoded for, or at least not in the same genomic locus. Dred_0143 is an A subunit of the Hdr protein, and is not localized in proximity of any HdrB or HdrC homologs. Only highly speculative hypotheses can be made about its function. It may, as has been suggested for other Hdrs in sulfate-reducing bacteria, be involved in sulfate reduction. Alternatively, HdrA could be an intermediate electron carrier in another pathway. In particular, it could be responsible for Fd reduction. If this were the case, one could propose that the energy requirement for this reaction could be obtained chemiosmotically, given its possible membrane localization, and invoke the involvement of Dred_2985, an H^+^ pyrophosphatase, putatively involved in energy conservation in *D. reducens* (Junier et al., 2010). A final possibility, is that this protein is capable of transferring electrons directly to Fe(III). In all these hypotheses, the likely electron donor for the reaction catalyzed by HdrA is NADH.

The last redox protein identified in the cell extracts is annotated as alkyl hydroperoxide reductase (AhpC type protein, Dred_1533). This is the only redox protein for which LC-MS/MS spectrum counts are higher in the protoplast extract from the Fe(III) reducing than the fermentation culture.

The function of AhpC-type proteins is to respond to the stress imposed by the presence of peroxides, by reducing them to water. It could be hypothesized that superoxide or hydroxyl radicals accumulate as a consequence of Fenton reactions occurring in the presence of iron ions, particularly Fe(II) (Touati, 2000). Radicals are eliminated by bacterial cells by dismutation to oxygen and hydrogen peroxide, which in turn can be eliminated by enzymes with peroxidase activity, such as Dred_1533 (Tally et al., 1977; Parsonage et al., 2008). However, it is not entirely clear how peroxide would be available for the Fenton reaction to occur under anoxic conditions.

Despite the presence of a protein domain related to AhpC (PF00578), it is not certain that Dred_1533 actually exhibits peroxide reductase activity. Interestingly, the Dred_1533 gene was found to be up-regulated also during U(VI) reduction, relative to fermentation and sulfate-reducing conditions (Junier et al., 2011). In the study investigating the transcriptome of *D. reducens* during U(VI) reduction, it was found that not only Dred_1533, but also the region adjacent to this gene is upregulated in the presence of U(VI). This region (Dred_1527–1533) comprises genes involved in cadmium resistance, ferric iron uptake (among which Dred_1529, a solute binding protein also found in our dataset, Table SI-5) and a *c*-type cytochrome biogenesis protein (Dred_1532), as well as the AhpC-type protein. The investigators also found some similarity between Dred_1533 and CcmG, a cytochrome maturation protein. This, and the genomic colocalization with Dred_1532, induced them to hypothesize a potential involvement of Dred_1533 in *c*-type cytochrome biosynthesis (Junier et al., 2011). Indeed, the Dred_1533 BLASTp best hits (all have significant alignments: *E*-value < 1e–28) are proteins involved in peroxide stress management, *c*-type cytochrome biosynthesis, or thiol-disulfide oxidoreduction. If this protein is involved in iron reduction, the latter is its most likely primary activity. Its apparent involvement in both Fe(III) and U(VI) reduction suggests that it is an important protein to consider for further study.

Amongst the transporter proteins, we identified proteins belonging to the HlyD family (Dred_0453 and Dred_3060, PF12700) and to the outer membrane efflux protein family (Dred_3141, PF02321), which includes *E. coli* TolC, for example (Benz et al., 1993; Johnson and Church, 1999; Pimenta et al., 2005). These two families comprise proteins that, in Gram-negative bacteria, constitute the type I secretion system, which is an ABC-type transporter, responsible for the export of protein, such as S-layer proteins and proteases, or toxins. In Gram-negative bacteria, HlyD is a periplasmic membrane fusion protein connecting the inner membrane export protein (HlyB) to the outer membrane export protein (TolC) and allowing the direct secretion of proteins from the cytoplasm to the extracellular environment (Delepelaire, 2004; Pimenta et al., 2005). The structure and function of this secretion complex may be similar in *D. reducens*, with appropriate modifications, as several proteases and S-layer proteins were identified in the surface layers extracts (Figure 5). A SecD/SecF-type protein (Dred_1669, PF02355 and PF07549) was also identified, which is predicted to be involved in protein export across the membrane (Schneewind and Missiakas, 2012).

Many proteases were identified in the surfaceome of *D. reducens*. Extracellular proteases exhibit various functions in bacteria. They play an important role in the cleavage of signal peptides for maturation and final localization of secreted pro-proteins (signal peptidase), and are also responsible for low-specificity protein degradation for uptake and use as protein synthesis building blocks (Wandersman, 1989). Other proteases are involved in peptidoglycan hydrolysis (Smith et al., 2000). Related to the latter function, we also found some PG hydrolases [e.g., proteins containing copper amine oxidase N-terminal domain (PF07833)], as well as proteins involved in PG synthesis (e.g., proteins containing penicillin binding domains). The proteins containing the PF07833 domain do not exhibit the catalytic domain of amine oxidases, but only the N-terminal domain, which is often found in cell wall hydrolases. In fact, some of these PF07833-containing proteins also contain other putative PG-hydrolyzing domains, such as glycoside hydrolase (PF00704) or peptidase family M23, which includes Gly-Gly endopeptidases (PF01551). Other PF07833-containing proteins either do not contain domains of known function or are likely to be involved in different functions: Dred_3143, for example, contains a metallo-ß-lactamase fold (PF0753) and thus could be involved in antibiotic resistance. The penicillin binding domain, instead, is known to be associated with membrane proteins responsible for polymerizing and cross-linking cell wall building blocks (Smith et al., 2000). Dred_0669, a protein identified to contain this domain, is a homolog of the *B. subtilis* sporulation protein SpoVD, which is involved in spore cortex synthesis (Liu et al., 2010). Since *D. reducens* cells were in the vegetative form when the surface proteins were extracted, it is possible that SpoVD in this organism is responsible for both vegetative and spore PG synthesis.

Overall, we have shown that *D. reducens* is capable of reducing extracellular Fe(III) with lactate as an electron donor by direct cell-TEA contact. However, the cells do not appear to conserve enough energy from this process to grow substantially. Our investigation of the surfaceome of *D. reducens* provided insights on the functions fulfilled by this cell-compartment, and allowed us to identify three enzymes, i.e., Dred_0143, Dred_0462, and Dred_1533, which could putatively be involved in Fe(III) reduction. However, further investigations are required to probe whether these proteins are truly involved in electron transfer to extracellular TEA. Unfortunately, the lack of genetic system provides limited opportunity to use a direct approach to tackle this question for *D. reducens*. In addition, the work revealed a considerable number of proteins (120) with functions in solute transport, signal transduction, proteolysis and chemotaxis as well as proteins of unknown functions with localization of the surface of the cell. The majority of these proteins are associated with the cytoplasmic membrane and likely extend into the cell wall and beyond, in some cases.

## Author contributions

Elena Dalla Vecchia conducted all Fe(III) reduction experiments, RNA and protein extractions, (q)RT-PCR, tDNA electrophoresis, SDS-PAGE and data analysis, and wrote the first draft of the manuscript; Paul P. Shao carried out TEM imaging for the identification of extracellular appendages; Elena Suvorova performed TEM and HR-TEM imaging of the products of Fe(III) reduction and SAED analysis; Romain Hamelin and Diego Chiappe were involved in the experimental design for the surfaceome extraction experiments, processed the protein extracts and carried out the LC-MS/MS analysis; Rizlan Bernier-Latmani is the PI of this study, is responsible for the design of most experiments, tightly collaborated with Elena Dalla Vecchia for data analysis, and proofread and edited the manuscript.

### Conflict of interest statement

The authors declare that the research was conducted in the absence of any commercial or financial relationships that could be construed as a potential conflict of interest.
