# Supplementary material for: Characterization of the surfaceome of the metal-reducing bacterium Desulfotomaculum reducens
Source: Front Microbiol. 2014 Aug 19;5:432. doi: 10.3389/fmicb.2014.00432 (PMC4137172; doi:10.3389/fmicb.2014.00432)
Supplement: Supplementary file 1 [file DataSheet1.ZIP › Data Sheet 1.DOCX]

**Characterization of the surfaceome of the metal-reducing bacterium *Desulfotomaculum reducens***

**Supplementary Information**

**Elena Dalla Vecchia^1^, Paul P. Shao^1+^, Elena Suvorova^1^, Diego Chiappe^2^, Romain Hamelin^2^, Rizlan Bernier-Latmani^1^***

^1^Environmental Microbiology Laboratory, Environmental Engineering Institute, École Polytechnique Fédérale de Lausanne, Lausanne, Switzerland.

^2^Proteomics Core Facility, Core Facility PTECH, École Polytechnique Fédérale de Lausanne, Lausanne, Switzerland.

*** Correspondence:** Prof. Rizlan Bernier-Latmani, EPFL ENAC IIE EML,CH A1 375, Station 6, 1015 Lausanne, Switzerland.

[rizlan.bernier-latmani@epfl.ch](mailto:rizlan.bernier-latmani@epfl.ch)

**Figure SI-1 Cell-free medium (empty circle), lysed (empty triangles) or killed cells (empty squares) are unable to reduce HFO in the presence of lactate as an electron donor. The individual curves are undistinguishable because they overlap. Vegetative cells positive control was plotted as a comparison (full diamonds).**

**Figure SI-2 (A) TEM image of a cell from a Fe(III)-citrate reducing culture with lactate and the EDS spectrum (in the inset) showing the presence of Fe containing precipitate on the cell. (B) TEM image, (C) selected area electron diffraction (SAED) pattern and (D) high resolution TEM (HRTEM) image of magnetite, Fe3O4, the product of HFO reduction with lactate as an electron donor. Inset in HRTEM image corresponds to a Fourier filtered image of the magnetite single crystal along the [432] direction. Samples whole mounts on C coated Au (A) or Cu (B, C, D) grids.**

**Table SI-2 qRT-PCR results: comparative expression of *nrf*A and *nrf*H relative to 16S rRNA under different conditions (2^(-∆Ct)) and relative to fermentation (2^(-∆∆Ct)).**

|  | ***nrfA*** | | ***nrfH*** | |
| --- | --- | --- | --- | --- |
| **Conditions** | **2^(-∆Ct)** | **2^(-∆∆Ct)** | **2^(-∆Ct)** | **2^(-∆∆Ct)** |
| **Fermentation** | 1.2E-04 | 1.0E+00 | 2.4E-05 | 1.0E+00 |
| **Fe(III)-citrate + lactate** | 8.6E-07 | 7.3E-03 | 8.2E-08 | 3.4E-03 |

**Figure SI-3 Expression of the two *c*-type cytochromes encoded in the genome of *D.reducens*, NrfA and NrfH: electrophoresis gel showing the product of RT-PCR for *nrf*A and *nrf*H: from left to right in duplicate lanes: *nrfA* in pyruvate fermentation, *nrfA* in Fe(III)-citrate reduction with lactate, *nrfH* in pyruvate fermentation, *nrfH* inFe(III)-citrate reduction with lactate.**

**
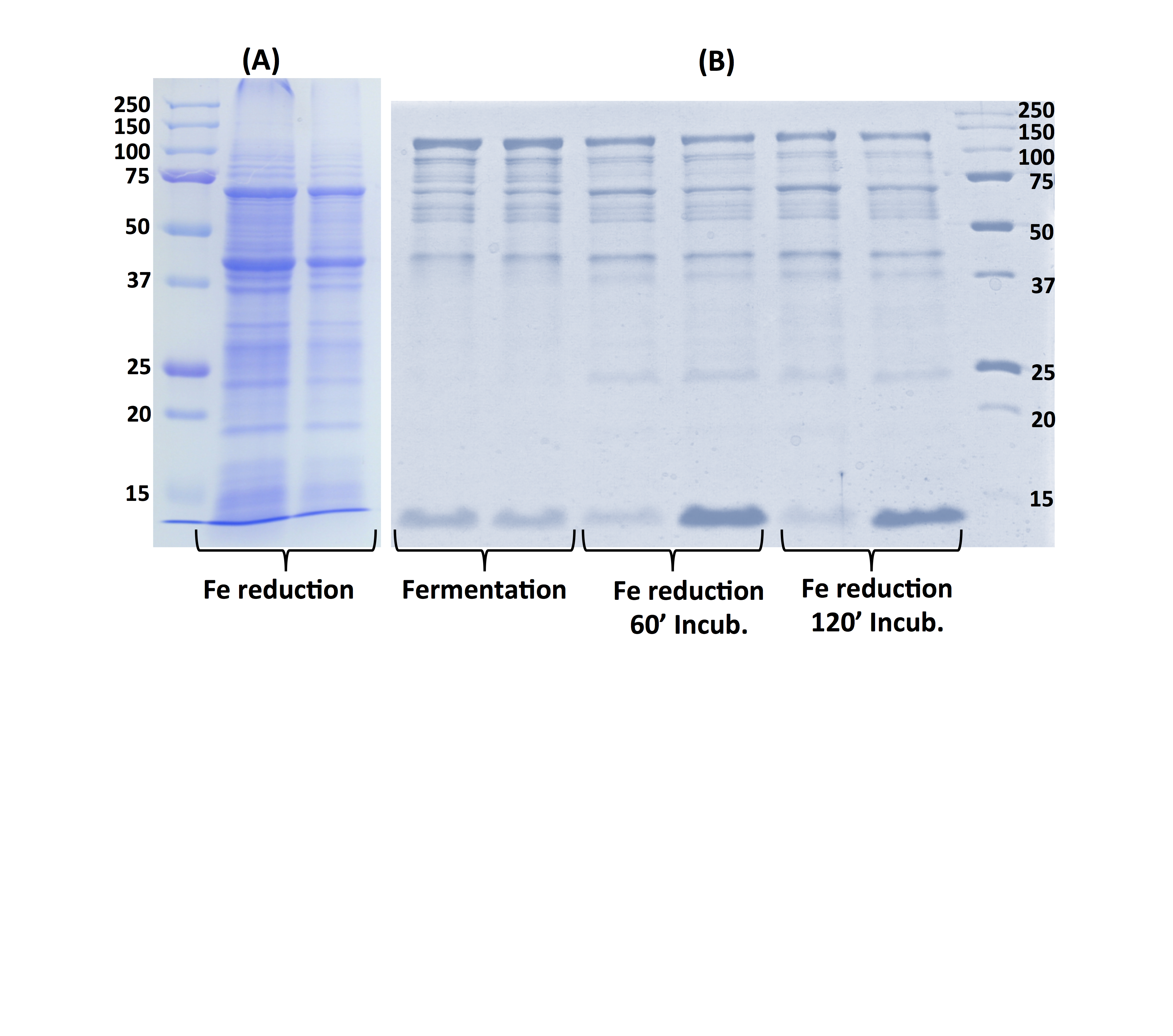
**

**Figure SI-4 SDS-PAGE of (A) total cell lysate of a Fe(III)-citrate reducing culture with lactate as the electron donor (duplicate lanes) and (B) of protein extracts from the protoplast experiment (duplicate lanes of pyruvate fermenting cells incubated 45 minutes, and Fe(III) reducing cells incubated 60 or 120 minutes in lysozyme). The thick band smaller than 15 kD is lysozyme. This figure indicates clearly that the profile of proteins extracted by lysis of the CW is significantly different from the total cell lysate and appears to be less diverse.**

**Figure SI-5 Multiples sequence alignment made with Clustal Omega (Sievers et al., 2011) of the two NarI-like (nitrate reductase, subunit gamma) identified in the surfaceome of *D. reducens*. Colored text indicates similar residues (red: small and hydrophobic residues (AVFPMILW); blue: acidic (DE); magenta: basic (RK); green: hydroxyl, sulfhydryl and amine residues (STYHCNGQ)). “*” indicates full residue conservation, “:” indicates conservation between groups of strongly similar properties, “.” indicates conservation between groups of weakly similar properties.**

**Table SI-1 Relative quantification between different conditions from the dimethyl-labelled samples obtained from cell shaving/shedding. These data were obtained by calculating the significance B values for each of the identified proteins using Perseus. In sheet “Legend” the content of each colums of sheet “SignificanceB_proteins” is described. This data is also uploaded on the PRIDE database (accession number: ), where proteins are identified with NCBI GI accession numbers: since there is some redundancy in the accession numbers (different GIs refer to the same protein), in sheet “protein ID synonyms” we listed all the possible accession numbers that could appear in reference to the same protein and grouped them by color.**

**Table SI-3 List of proteins identified in the protoplast experiment prior to screening for surface proteins. In sheet “scaffold order” proteins are listed in order of decreasing level of expression, and their predicted molecular weight as well as their normalized quantitative value in each duplicate sample (i.e., Fe(III) reduction incubated 60 or 120 min, and fermentation) is indicated. Cells shaded in yellow indicate surface localization, in green cytoplasmic localization (excluded from following analysis). Sheet “cytoplasmic vs surface” contains the exact same information, but proteins are sorted by cellular localization.**

**Table SI-4 List of proteins identified in the shaving/shedding experiment prior to screening for surface proteins. This list was derived from the dataset in Table SI-1 filtered in Scaffold with a FDR of 1 %. In sheet “scaffold order” proteins are listed in order of decreasing level of expression and their predicted molecular weight is indicated. The values indicated in the “quantitative value” section do not distinguish between the mixed labeled samples, thus they are not really meaningful and should not be considered as quantitative. The purpose of this list is only to indicate the dataset used for subsequent screening for surface proteins: cells shaded in yellow indicate surface localization, in green cytoplasmic localization (excluded from following analysis). Sheet “cytoplasmic vs surface” contains the exact same information, but proteins are sorted by cellular localization.**

**Table SI-5 List of proteins identified in the surfaceome of *D. reducens* divided by general function (transport, chemotaxis, proteases and CW hydrolases, other or unknown function, redox). For each protein the following information is provided: its locus tag, its annotation, its molecular weight (MW), its function, its putative localization (CM = cytoplasmic membrane, CW = cell wall, S-U = surface-undefined, EC = extracellular, SL= S-layer, C = cytoplasm), its localization predicted by PSORTb (as putative localization and in addition: S= surface: not cytoplasmic, U = unknown) and LocateP, the number of transmembrane helices (#TMH) and its topology as predicted by (TMHMM), the experiment in which this protein was identified (P = protoplast formation experiment, Sh = Shaving experiment, P&Sh = both experiments); for the proteins identified in P, the average spectrum counts and error (obtained from the label-free quantitative method (normalized total spectra) of Scaffold 4.1.1) for the fermentation and the Fe(III) reduction samples are also indicated; for the proteins identified in Sh, a comment is included if a protein is more expressed in one condition than the other. Green cells indicate proteins more expressed in fermentation relative to Fe(III) reduction according to one of the two experiments, orange cells indicate the opposite.**

**References**

Sievers, F., Wilm, A., Dineen, D., Gibson, T. J., Karplus, K., Li, W., Lopez, R., McWilliam, H., Remmert, M., Söding, J., et al. (2011). Fast, scalable generation of high-quality protein multiple sequence alignments using Clustal Omega. *Mol. Syst. Biol.* 7. doi:10.1038/msb.2011.75.
